# Compatibility and Efficacy of *Isaria fumosorosea* with Horticultural Oils for Mitigation of the Asian Citrus Psyllid, *Diaphorina citri* (Hemiptera: Liviidae)

**DOI:** 10.3390/insects8040119

**Published:** 2017-10-31

**Authors:** Vivek Kumar, Pasco B. Avery, Juthi Ahmed, Ronald D. Cave, Cindy L. McKenzie, Lance S. Osborne

**Affiliations:** 1 Mid-Florida Research and Education Center, Institute of Food and Agricultural Sciences, University of Florida, 2725 South Binion Road, Apopka, FL 32703, USA; lsosborn@ufl.edu; 2 United States Department of Agriculture, Agricultural Research Services, 2001 South Rock Road, Fort Pierce, FL 34945, USA; cindy.mckenzie@ars.usda.gov; 3 Indian River Research and Education Center, Institute of Food and Agricultural Sciences, University of Florida, 2199 South Rock Road, Fort Pierce, FL 34945, USA; pbavery@ufl.edu (P.B.A.); juthi12@ufl.edu (J.A.); rdcave@ufl.edu (R.D.C.)

**Keywords:** entomopathogenic fungi, mineral oil, adjuvants, citrus pest, fungal development index

## Abstract

Horticultural oils are an important component of integrated management programs of several phytophagous arthropods and pathogens affecting fruit, ornamentals and vegetables in greenhouse and field production systems. Although effective against the target pest, their incompatibility with biological control agents can compromise efforts to develop eco-friendly management programs for important agricultural pests. In this study, we assessed the in vitro effect of selected refined petroleum oils used in citrus and other horticultural crops with a biopesticide containing the entomopathogenic fungi, *Isaria fumosorosea* (PFR-97) under laboratory conditions. Further, we used leaf disk bioassays to evaluate the combined efficacy of petroleum oils and *I. fumosorosea* against the Asian citrus psyllid, *Diaphorina citri* (Hemiptera: Liviidae), a major pest of citrus in the United States. All five petroleum oil treatments (Orchex, Sun Pure, Conoco Blend -1, Conoco Blend -2, and JMS) were compatible with *I. fumosorosea* blastospores, as none of them were found to affect *I. fumosorosea* colony-forming units and radial fungal growth measured at 3, 6, 9, and 12 days post-inoculation. All mixed treatments performed better than *I. fumosorosea* alone against *D. citri*, where the highest mean survival time of *D. citri* was 12.5 ± 0.7 days. No significant differences in *D. citri* survival time and *I. fumosorosea* growth (fungal development index) on dead cadavers, which is important for determining their horizontal transmission, were observed when mixed with Orchex, Sun Pure, Conoco Blend -2, and JMS. Results indicated that horticultural oils in combination with *I. fumosorosea* could offer citrus growers an alternative treatment for integrating into their current management programs while battling against *D. citri* in citrus production systems. Due to their eco-friendly, broad-spectrum effect, it could provide control against various citrus pests, while also encouraging the retention of effective chemistries for a longer period in the marketplace. However promising, these combination treatments need to be tested further with *I. fumosorosea* under grove conditions to confirm their field efficacy.

## 1. Introduction

Biological control of arthropod pests using entomopathogenic fungi has been practiced for over 100 years, but with recent growing demands to search for eco-friendly alternate management practices, they have regained impetus among researchers, growers, and pest-management professionals for potential use in sustainable pest-management programs [1,2]. Given favorable growing conditions, they can act against a variety of pestiferous species of agricultural importance and can ensure minimal risk for non-target organisms, including beneficials such as bees, earthworms, parasitoid wasps, and predatory beetles [3]. Their unique mode of action against the pest by means of cuticular invasion or ingestion *per os* can result in arthropod death due to a variety of factors, which can be accounted for by any of the following, or a combination after application: (1) mechanical damage from tissue invasion, (2) depletion of nutritional content inside the insect body, and/or (3) production and spread of toxins [2]. In addition, their self-multiplicative ability on the substrate, which assists in their horizontal transmission, gives them an edge over the traditional synthetic insecticides, and ensures longer persistence and prolonged pest control. However, their performance in the field depends greatly upon abiotic conditions, forcing growers to use them often in conjunction with other pesticides to achieve adequate pest control [4]. Since infection by entomopathogenic fungi requires cuticular contact with the pest, their efficacy can be enhanced through the use of other agricultural chemicals, such as emulsifiers, adjuvants, horticultural oils to ensure better homogeneity while in suspension, dispersal of propagules, and the protection of spores against unfavorable weather parameters.

In Florida, citrus is considered a premier commodity crop, which generates about $9 billion annual revenue [5,6]; thus, the state’s economy, as well as its labor/work force, greatly depends upon the citrus industry thriving. Recently, the Asian citrus psyllid, *Diaphorina citri* Kuwayama (Hemptera: Liviidae), has emerged as a major biological factor affecting citrus production. *Diaphorina citri* is a pest of Asian origin, and a vector of one of the most destructive pathogens of citrus, ‘*Candidatus* Liberibacter’ species, which is a phloem-limited bacterium associated with citrus greening disease, or ‘huanglongbing’ (HLB), worldwide [7,8]. In Florida, the fruit and farming industry has taken a direct hit, reporting a 23% decline in cumulative citrus production and an associated 48% job losses within five years of the detection of HLB disease in the state [8]. In a recent report, USDA-NIFA [6,9] forecasted a further decline in citrus production for Florida of over a 70% reduction compared to 1997–1998 production levels. Considering the severity of damage and economic loss associated with this disease, current control strategies rely heavily on the use of chemical insecticides for the control of the vector. Although a range of synthetic chemical insecticides is effective against the psyllid [10,11,12], recent studies suggest that these intensive psyllid control programs have not been successful in mitigating the spread of HLB disease [7,13]. Excessive reliance on the selective insecticides for the control of psyllids has resulted in reduced susceptibility of the pest against a variety of insecticides, including pyrethroids, carbamates, organophosphates and neonicotinoids [14,15]. A chemically driven psyllid management program could impact beneficial populations present in the groves, and thwart the parasitic wasp-based classical biological control programs being implemented for *D. citri* [16,17]. Furthermore, there are challenges in the application of chemical insecticides for the residential trees and abandoned citrus groves that serve as a reservoir of HLB disease. 

Considering the importance of alternative low-risk insecticides for psyllid control, researchers have evaluated horticultural mineral oils (HMOs, refined petroleum oil) and entomopathogenic fungi for their potential integration in citrus management programs [4,12,18,19,20,21]. Qureshi et al. [12] found that refined petroleum oil, when used alone, did not provide adequate suppression of *D. citri,* because the mean adult and nymphal suppression was only 36% and 50%, respectively, for <3 weeks. When used as an adjuvant, HMO improved the efficacy of synthetic chemical insecticides compared to insecticides applied without HMO. Furthermore, HMO provided control for additional citrus pests including leafminer and rust mites. Among the entomopathogenic fungi, the utility of *Isaria fumosorosea* Wise against *D. citri* has been reported by several authors in Florida [4,20,21,22,23,24]. However, the potential impact of agrochemicals such as HMO routinely applied in citrus production has not been broadly investigated. Therefore, considering this lack of information on the interaction of HMO with *I. fumosorosea*, in the current study, we assessed the compatibility of selected refined petroleum oils with *I. fumosorosea* and their combined effect on survivorship of *D. citri* adults. We believe the outcome of this study will help promote the adoption of *I. fumosorosea*-based eco-friendly management programs of *D. citri* and other citrus pests [24] and improve their potential role in preventing insecticide resistance. 

## 2. Materials and Methods 

### 2.1. Insects

*Diaphorina citri* adults used in these experiments were obtained from the USDA-ARS laboratory colony previously described by Hall et al. [25] and established during early 2000 at the U.S. Horticultural Research Laboratory (USHRL) in Fort Pierce, Florida (FL). Originally collected from citrus, the psyllids had been continuously reared on orange jasmine, *Murraya paniculata* (L.), until March 2010, when *Citrus macrophylla* Wester was substituted as the rearing plant for reasons related to the availability of plants. Colonies were housed in Plexiglas (0.6 × 0.6 × 0.6 m) or BugDorm-2 cages (MegaView Science Education Services Co., Ltd., Taichung, Taiwan) under the following environmental conditions: 20–28 °C, 40–80% RH and a 14 h light (L): 10 h dark (D) photoperiod. There had been no field-collected psyllids added to the original colony since establishment. The colony is maintained by transferring adults to new plants using procedures similar to those described by Skelley and Hoy [26], and is routinely tested to ensure that the colony remains free of ‘*Candidatus* Liberibacter’. 

### 2.2. Preparation of Emulsions with I. fumosorosea

The fungal suspension of *I. fumosorosea* Apopka 97 strain was made by mixing 1 g of PFR-97 20% WDG (Certis USA, Columbia, MD, USA) to 100 mL of distilled water with a magnetic stirring bar for 30 min and then allowed to settle for 30 additional minutes for the inert material to precipitate out of the suspension, leaving the supernatant. The supernatant was then adjusted to 10^7^ blastospores/mL by using a C-Chip disposable improved Neubauer hemocytometer (Incyto Co., Ltd., Chungnam-do, Korea), which was used to make all the emulsions. A 3% oil to water emulsion was made by pipetting 300 µL of oil into a 15 mL sterile disposable Fisherbrand^®^ (Thermo Fisher Scientific, Hampton, NH, USA) plastic centrifuge tube containing 8.70 mL of distilled water. Next, a 1 mL aliquot of the previously mixed fungal suspension of PFR-97 (10^7^ blastospores/mL) was added to the tube. The tube was capped and vortexed 5 times for 15 s. The final concentration of *I. fumosorosea* for each emulsion was 10^6^ blastospores/mL in each experiment. Blastospore viability was assessed to be 100% for all experiments conducted. This procedure was followed for all the HMOs tested (Table 1).

### 2.3. Compatibility of I. fumosorosea with HMOs In Vitro

**Figure 1:**
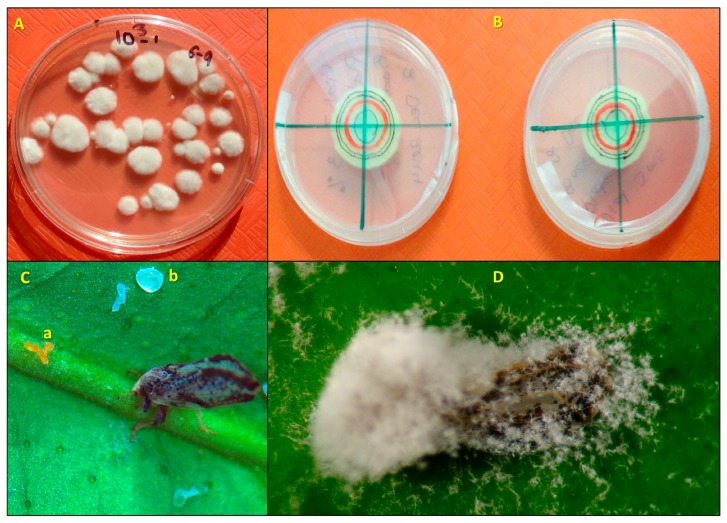
Viability and infection parameters of *Isaria fumosorosea* on the Asian citrus psyllid, *Diaphorina citri* alone and after being mixed with horticultural mineral oil (HMO) treatments: (**A**) *I. fumosorosea* CFUs growing on potato dextrose agar; (**B**) radial growth of *I. fumosorosea* with HMO (left) compared to control (right); (**C**) *D. citri* adult previously having fed on the midrib vein of a citrus leaf disk inside of the bioassay chamber, which is now dead, infected with *I. fumosorosea* and attached to the leaf by the fungus growing through the tarsi; (**a**) eggs laid previous to adult psyllid death are now infected; (**b**) honeydew droplet deposited after feeding inside bioassay chamber; (**D**) Colonization of adult *D. citri* by fungus and production of conidia on the phialides extending away from the infected wings onto the leaf; note the fungus horizontal growth from the mycosed insect into the surrounding area.

The compatibility of a blastospore formulation of *I. fumosorosea* with different HMOs was evaluated under sterile laboratory conditions. The effects of test materials on in vitro colony-forming units (CFUs) and radial growth rates of *I. fumosorosea* were assessed using two different techniques. First, all test suspensions and *I. fumosorosea* alone (control) were prepared as described above, in sterile 15 mL centrifuge tubes with *I. fumosorosea* (10^6^ blastospores/mL). All tubes were mixed thoroughly by vortexing for 15 s (5×) prior to use in both experiments. Petri dishes (100 × 15 mm) containing Difco™ potato dextrose agar (PDA: 3.9% *w*/*v*, Difco Laboratories, Detroit, MI, USA) with streptomycin (0.5%) added were used in both experiments. To determine the in vitro effect of the HMOs on the number of *I. fumosorosea* CFUs, each treatment emulsion suspension was serial diluted to 10^3^ blastospores/mL, and a then a 100 µL droplet of each treatment suspension was spread on each of 5 replicate PDA plates using a flame-sterilized glass spreader. Plates were sealed with Parafilm^®^ (Bemis Company, Inc., Neena, WI, USA) and transferred to a growth chamber maintained at 25 °C, under a 14 h photophase. After 7–10 days incubation, the CFUs were counted (Figure 1A). To determine the in vitro effect of the HMOs on the radial growth rates of *I. fumosorosea*, a 20 µL droplet of each treatment suspension was inoculated in the center of the PDA dish. Plates were sealed with Parafilm^®^ and transferred to a growth chamber maintained the same as above. After 4 days, the first original colony diameter (mm) was traced on the dish bottom and recorded as day 0, and then each new diameter at 3, 6, 9, and 12 days post-inoculation was measured both horizontally and vertically from the original diameter (Figure 1B). The differences in diameters assessed at 3, 6, 9 and 12 days from the original were recorded. For both techniques, there were 5 replications per treatment, and the study was repeated on separate days. 

### 2.4. Compatibility of I. fumosorosea with Spray Oils In Vivo

The compatibility of a blastospore formulation of *I. fumosorosea* with different HMOs against the adult psyllid was evaluated using a leaf disk bioassay under optimum conditions in the laboratory. Adult psyllids (mixed population of males and females) 7–14 days old were obtained from the colony reared at the USHRL (see details above) in screw-top glass vials, brought back to the laboratory at the University of Florida, Indian River Research and Education Center (IRREC), Ft. Pierce, FL and kept on the lab bench (23–25 °C) for a period of 24 h before using them in the experiment. The ratio of males to females was approximately 1:1 in the colony. 

Duncan grapefruit (*Citrus paradisi* Macf.) seedlings were grown in Premier Pro-mix General Purpose Growing Medium from seed in size C10 ‘Cone-tainers’™ (Stuewe & Sons, Inc., Corvalis, OR, USA) for approximately 6 months. Leaves of similar age and size were excised from seedlings grown in the USHRL greenhouse, placed in resealable plastic bags, and brought back to the laboratory at IRREC. The leaves were washed gently in tap water containing liquid soap (1 drop of soap/11.4 L of water) to remove any possible saprophytic fungi present on the surface, then rinsed three times with water to remove any soap residue, and placed on brown paper towels on the lab bench until dry. Leaf disks (~962 mm^2^) were punched using a cork borer from clean citrus leaves and the abaxial surface (underside) was sprayed using a Nalgene pump sprayer with 200 µL of either *I. fumosorosea* blastospore suspension alone (control) or mixed with each HMO. 

Ten Petri dishes (35 × 10 mm) containing 1% water agar were prepared modified as described by Hall and Nguyen [16] for each treatment and the controls in order to preserve the leaf disks and maintain a semi-constant RH percentage for the duration of the experiment. The modification consisted of placing each leaf disk on top of the solidified agar in the dish, abaxial side facing up. The experiments were repeated on two separate dates for a total of 20 disks per treatment (both experiments combined together). Treatment suspensions and water on leaf disks were allowed to air dry inside the Petri dishes containing water agar with the abaxial side of the leaf facing up. One randomly chosen psyllid was transferred using a camel hair brush and allowed to walk off of the brush onto the dish top. Separate brushes were used for each treatment when transferring the psyllid to prevent cross contamination. The bioassays, now called arena chambers were sealed with Parafilm™ and incubated in a growth chamber at 25 °C under a photoperiod of 14 h L: 10 h D for a 1 week period. All arena chambers were sealed for the first 24 h to maintain a high relative humidity (~100%) and promote germination of the fungus. Sealed arena chambers, one for each treatment, were randomized and arranged in 5 rows on two separate plastic orange cafeteria trays (45.7 × 30.5 × 2.0 cm; Winco Mfg., LLC, Ocala, FL, USA). Both trays were placed in the growth chamber on separate racks and incubated for 24 h. After 24 h, the arena chambers were then unsealed, and individual adult psyllids were assessed for mortality. On a daily basis after assessment, each arena chamber was placed back into the original row on the tray and each tray was switched from the top to the lower rack in the growth chamber. Each disk (Petri dish) was counted as an independent replicate within a treatment group (10 disks/treatment), and each of the treatments was assessed on two separate dates (*n* = 20/treatment). To investigate whether oils impacted fungal growth development post-mortem, psyllid cadavers were examined daily at 40×, and individuals were scored according to the fungal development index (FDI) assessment proposed by Avery et al. [22], e.g., 1.0 (no symptoms; Figure 1C); 1.5 = appearance of fungal hyphae through the exoskeleton of the psyllid body; 2.0 = hyphae protruded through head, thorax, wings of host; 2.5 = initial conidia formed on host; 3.0 = fungus has colonized and formed conidia on all sections of the psyllid body (Figure 1D).

### 2.5. Statistical Analysis

Treatments from laboratory tests were compared through one-way ANOVA conducted on the data and post-hoc means separated where appropriate through Tukey’s HSD test at *p* < 0.05. All statistical analyses were conducted using SAS Proc GLM procedures and executed on a PRO _WIN 6.1 platform (SAS 2002–2012; SAS Institute Inc., Cary, NC, USA). The median lethal survival time (ST_50_) for 50% of the adult psyllids in the insect bioassays per treatment was also compared through Kaplan Meier survival analysis followed by a log rank test (SAS JMP 8 for Windows 2013). Mean survival for the psyllids and the FDI per treatment was compared with the untreated control by using the Tukey’s HSD test (*p* < 0.05).

## 3. Results

### 3.1. Compatibility of I. fumosorosea with HMOs In Vitro

The number of CFUs and radial growth rate of *I. fumosorosea* alone and mixed with HMOs at different sampling dates are presented in Table 2. Mean number of CFUs per plate ranged 29.9 ± 1.29–42.6 ± 3.18, and no significant negative impact of oils was observed on *I. fumosorosea* blastospore viability. A significantly higher number of CFUs was observed with Conoco Blend -2 compared to *I. fumosorosea* (control) and Sun Pure treatments. Comparisons of the effects of oils on *I. fumosorosea* colony radial growth revealed no apparent impact until 9 days post-inoculation. The radial colonial hyphal growth rate in the JMS oil treatment was significantly higher than the rate in the Conoco Blend -2 on all the sampling dates. Maximum radial colonial growth rates for the different treatments ranged 16.8 ± 0.68–19.4 ± 0.48 mm by the end of the study. Fungal spore growth on PDA and the adult psyllid body is shown in Figure 1.

### 3.2. Compatibility of I. fumosorosea with HMOs In Vivo 

**Figure 2:**
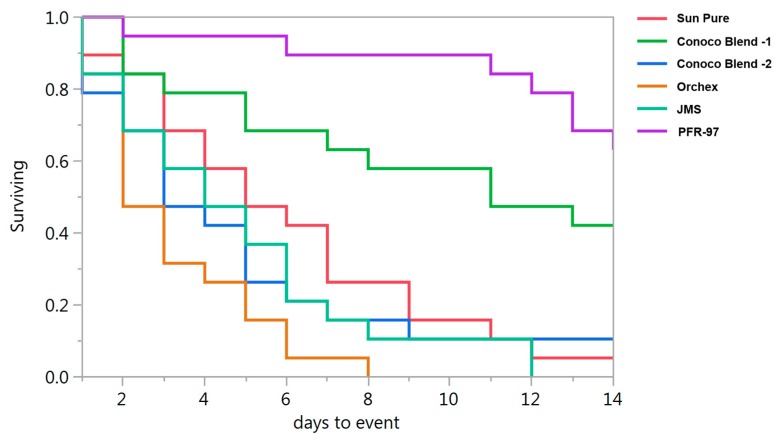
Survival curves for *Diaphorina citri* adults after being exposed to *Isaria fumosorosea* (PFR-97) alone or mixed with different HMOs.

**Figure 3:**
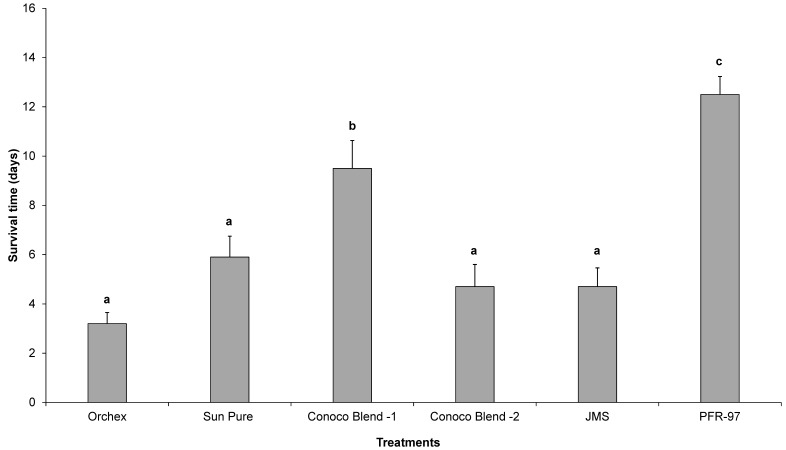
The mean number (± SEM) of days’ survival for *Diaphorina citri* adults after being exposed to *Isaria fumosorosea* (PFR-97), alone or mixed with different HMOs. Letters located above the SEM bars that are not the same are significantly different (Tukey’s HSD test, *p* < 0.05).

**Figure 4:**
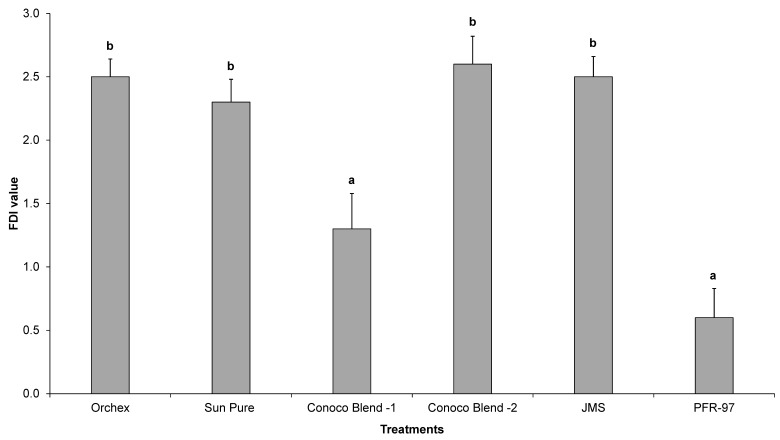
The mean Fungal Development Index (FDI) ± SEM value for *Diaphorina citri* adults after being exposed to *Isaria fumosorosea* (PFR-97), alone or mixed with different HMOs. Letters located above the SEM bars that are not the same are significant different (Tukey’s HSD test, *p* < 0.05).

Psyllid survival estimates indicated that the HMO treatments improved the efficacy of *I. fumosorosea*. According to the Kaplan-Meier survival analysis, ~75% of psyllids were surviving 14 days post-treatment application, whereas 100% psyllid mortality was observed with Orchex and JMS treatments on days 8 and 12, respectively (Figure 2). The ST_50_ for psyllids in Orchex was 3.15 ± 0.45 days versus 12.57 ± 0.77 days in *I. fumosorosea* alone (log rank *χ*^2^ = 55.6, *p* < 0.0001, df = 5). All the oil treatments significantly reduced the survival time of psyllids compared to *I. fumosorosea* alone, and no significant differences in the mean survival time of psyllids were observed amongst oil treatments, except for Conoco Blend -1 (*F* = 27.22; df = 5, 90; *p* < 0.0001) (Figure 3 and Figure 4). Comparisons of the FDI values for dead psyllid cadavers observed over a 14 day period revealed that, for all the oil treatments except for Conoco Blend -1, the fungus colonized the psyllid significantly (*F* = 18.46; df = 5, 90; *p* < 0.0001) faster than *I. fumosorosea* alone (Figure 4). FDI values ranged between 2.6 ± 0.16 for Conoco Blend -2 treatment versus 0.6 ± 0.23 with *I. fumosorosea* alone.

## 4. Discussion

*Isaria fumosorosea* has been isolated from mycosed *D. citri* on the foliage of orange trees in Polk County, Florida [27], suggesting that this naturally occurring fungus should be tested under different abiotic and biotic parameters to determine if it can be promoted as a good microbial candidate for citrus pest management. As of 2017, there are two blastospore strains (Apopka 97 and ARSEF 3581) of *I. fumosorosea* available for research, of which only the Apopka 97 strain contained in the formulated product PFR-97 20% WDG is commercially available for use in field/food crops, including citrus [24]. Thus, it is imperative to screen it at various variables before recommending it to growers. The use of refined petroleum oils as an adjuvant is one such test. Among several advantages of using *I. fumosorosea* (Apopka 97 strain) against *D. citri* is its adaptability to the native climatic conditions (temperature 24–30 °C + high humidity) and efficacy against a wide range of citrus pests, including psyllids, mealybugs, leafminers and plant bugs [24].

There are three kinds of interactions (antagonistic, synergistic or neutral) that can be expected when a chemical is mixed with entomopathogenic fungi [4,28,29,30,31,32]. In our current study, we did not observe any negative impact by the petroleum oils on the growth parameters, i.e., CFUs and radial diameter estimates of *I. fumosorosea*. Rather, a synergistic effect on the potency of *I. fumosorosea* with Orchex and JMS was observed (Figure 2 and Figure 3). Similar results for the impact of oils on entomopathogenic fungal activity against hemipteran pests have been reported previously [4,31,32]. In addition, the FDI value for the Orchex, Conoco Blend -2, and JMS treatments reached beyond 2.5 by the end of the study period, and was significantly higher than the value in *I. fumosorosea* (0.6 ± 0.23) treatment alone. This indicates that the tested spray oils promote the hyphal growth of fungus and may help colonize on the substrate or insect cuticle, as well as increase the potential fungal horizontal transmission to the conspecifics in the sprayed regions. Although we did not identify the mechanism behind the additive effect of the HMOs on the activity of *I. fumosorosea*, we hypothesize that it could be due to improved adherence of the blastospores on the lipophilic psyllid surface. 

In the present study, we did not compare the impact of *I. fumosorosea* on the efficacy of HMOs against the adult psyllid. However, in a related study conducted under similar conditions [4], combination treatment of *I. fumosorosea* and oils significantly reduced the survivability of psyllid adults when compared to oils applied alone. In another study, Cuthbertson and Collins [32] reported that a significantly higher mortality of the second instar larvae of another hemipteran pest, *Bemisia tabaci* (Gennadius), was observed after application of mixed treatments of petroleum horticultural oil, Tri-Tek, and the entomopathogenic fungus *Beauveria bassiana*, than for each of the treatments applied alone under glasshouse conditions. These studies together suggest that there is potential in using HMOs and entomopathogenic fungi in an integrated management program for invasive pests; nonetheless, the compatibility of the selected products must be assessed and ensured before testing them in production systems.

Because citrus is a very high value crop, we do acknowledge the limitations of using entomopathogenic fungi over chemical insecticides for psyllid control, especially in regions where HLB has been detected previously. It takes about two days for *I. fumosorosea* to infect and kill *D. citri* adults [4] under controlled conditions, whereas acquisition and transmission of HLB by *D. citri* may occur within 24 h post-feeding [33,34,35]. In such a scenario, growers do not have many options other than selecting pesticides that provide instantaneous control of pests to minimize the risk of spreading disease. Thus, we speculate that the results of the current study may not be readily transferable to Florida citrus growers battling psyllid problems in their field, as there are additional factors that could affect fungal efficacy under field conditions; nevertheless, they could be useful elsewhere, where HLB is not a major issue. However, our results are encouraging for the concerted efforts of researchers involved in improving the utility of entomopathogenic fungi in abandoned citrus groves and residential trees (acting as HLB reservoir), where chemical control of citrus is problematic and not economical for the growers. Currently, several researchers in Florida and elsewhere are working towards finding effective tools to mitigate psyllids in such areas, and this work is an additional step in that direction. Furthermore, we believe that the outcome of the study will be important for growers engaged in the citrus nursery businesses, and controlled citrus production systems also known as CUPS (Citrus Under Protective Screen), where, apart from psyllids, other soft-bodied arthropods such as thrips, leafminers, and citrus mites are the major issues.

## 5. Conclusions

Considering the importance of alternative management practices and insecticide resistance management for citrus pests, the current study highlights three major findings: (1) selected HMOs are compatible with the entomopathogenic fungus *I. fumosorosea* at applied rates; (2) as an adjuvant, they improve the efficacy of *I. fumosorosea* against *D. citri* adults; and, most importantly, (3) they enhance the FDI value on dead cadavers, which is important for the existence of the entomopathogenic fungus in the affected region for longer durations as a result of the horizontal transmission of the spores to conspecifics, as well as other citrus pests. Our data provides additional support for using HMOs in combination with entomopathogenic fungi (mycoinsecticides); however, each HMO needs to be tested and evaluated for compatibility before being applied under field conditions. Including a mixed treatment of HMO and *I. fumosorosea* into the spray rotation, especially in the early spring or late fall, may enhance psyllid suppression without introducing a selection agent at the same time, and thus potentially provide the growers with an additional management tool. Future studies will focus on evaluating the residual efficacy of the tank mix suspension of HMOs and *I. fumosorosea* under field and CUPS for control of citrus pests.

## Figures and Tables

**Table 1:** Details of the emulsifier and entomopathogen used in different studies.

Product Name	Manufacturer	Description of Product	Active Ingredient (by wt.)	Final Concentration (*v*/*v* or *w*/*v*)
Orchex 796	Calumet Lubricants Co.	Petroleum Oil	99.2%	3%
Sun Pure Spray Oil 435	ConocoPhillips Co.	Petroleum Oil	99%	3%
Conoco Blend Spraybase 435 Oil	ConocoPhillips Co.	Petroleum Oil	100% *	3%
Conoco Blend Spraybase 435 Oil	ConocoPhillips Co.	Petroleum Oil	90%	3%
JMS Stylet Oil	JMS Flower Farms, Inc.	Petroleum Oil	97.1%	3%
PFR-97	Certis USA	*Isaria fumosorosea*	20%	1%

* In order to differentiate between two formulations, Conoco Blend with 100% mineral oil has been considered Blend -1 and with 90% ai as Blend -2.

**Table 2:** The mean number (±SEM) of colony forming units (CFUs) and radial colonial hyphal growth (mm) of *Isaria fumosorosea* (PFR-97), alone or mixed with various HMO treatments at 25 ± 1 °C, 60–80% RH, under a 12:12 h L:D photoperiod assessed in vitro over 12 days.

Treatment	Number of CFU’s	Radial Colonial Hyphal Growth (mm)/Days Post-Inoculation			
		3	6	9	12
Orchex	37.0 ± 1.4abc	4.9 ± 0.2ab	9.4 ± 0.2ab	14.2 ± 0.3b	19.0 ± 0.4b
Sun Pure	29.9 ± 1.3a	5.0 ± 0.4ab	9.8 ± 0.4b	14.0 ± 0.4b	19.2 ± 0.3b
Conoco Blend -1	36.9 ± 2.1abc	5.0 ± 0.2ab	9.3 ± 0.2ab	13.7 ± 0.4b	18.5 ± 0.4ab
Conoco Blend -2	42.6 ± 3.2c	4.0 ± 0.4a	7.9 ± 0.6a	11.9 ± 0.6a	16.8 ± 0.7a
JMS	40.0 ± 3.3bc	5.4 ± 0.3b	9.6 ± 0.4b	14.4 ± 0.4b	19.4 ± 0.5b
PFR-97 (water)	32.3 ± 2.4ab	5.0 ± 0.2ab	8.9 ± 0.3ab	13.7 ± 0.5b	18.5 ± 0.4ab
Statistical analysis	*F* = 4.41;	*F* = 2.42;	*F* = 3.19;	*F* = 4.48;	*F* = 4.28;
	df = 5, 45;	df = 5, 95;	df = 5, 95;	df = 5, 95;	df = 5, 95;
	*p* = 0.0024	*p* = 0.0413	*p* = 0.0105	*p* = 0.0010	*p* = 0.0015

Means values followed by different letters in a column are significantly different (Tukey’s test, *p* < 0.05).

## References

[1] Faria M.R., Wraight S.P. (2007). Mycoinsecticides and mycoacaricides: A comprehensive list with worldwide coverage and international classification of formulation types. Biol. Control.

[2] Sandhu S.S., Sharma A.K., Beniwal V., Goel G., Batra P., Kumar A., Jaglan S., Sharma A., Malhotra S. (2012). Myco-biocontrol of insect pests: Factors involved, mechanism, and regulation. J. Pathog..

[3] Lacey L.A., Grzywacz D., Shapiro-Ilan D.I., Frutos R., Brownbridge M., Goettel M.S. (2015). Insect pathogens as biological control agents: Back to the future. J. Invertebr. Pathol..

[4] Avery P.B., Pick D.A., Aristizábal L.F., Kerrigan J., Powell C.A., Rogers M.E., Arthurs S.P. (2013). Compatibility of *Isaria fumosorosea* (Hypocreales: Cordycipitaceae) blastospores with agricultural chemicals used for management of the Asian citrus psyllid, *Diaphorina citri* (Hemiptera: Liviidae). Insects.

[5] National Agricultural Statistics Summary (2013). Florida Agricultural Statistics 2011–2012.

[6] Iriarte F., Dewdney M., Johnson E., Paret M., Martini X., Andersen P., Small I., Loverstrand E., Nguyen N. Causal Organism: *Candidatus* Liberibacter asiaticus Insect Vector: Asian Citrus Psyllid *Diaphorina citri* Kuwayama. http://plantpath.ifas.ufl.edu/u-scout/Alert_files/CITRUS-HUANGLONGBING-PanhandleDiseaseAlert-FINAL-1-26-17-2.pdf.

[7] Hall D.G., Richardson M.L., El-Desouky A., Halbert S.E. (2012). Asian citrus psyllid, *Diaphorina citri*, vector of citrus, huanglongbing disease. Entomol. Exp. Appl..

[8] Hodges A.W., Spreen T.H. Economic Impacts of Citrus Greening (HLB) in Florida. http://www.crec.ifas.ufl.edu/extension/greening/PDF/FE90300.pdf.

[9] USDA NIFA in IPM in the South Citrus Greening Continues to Thwart Citrus Production in Florida. https://ipmsouth.com/2017/03/15/citrus-greening-continues-to-thwart-citrus-production-in-florida/.

[10] Qureshi J.A., Stansly P.A. (2008). Rate, placement and timing of aldicarb applications to control Asian citrus psyllid, *Diaphorina citri* Kuwayama (Hemiptera: Psyllidae), in oranges. Pest Manag. Sci..

[11] Stansly P., Arevalo H.A., Zekri M. (2010). Area-wide psyllid sprays in southwest Florida: An update on the cooperative program aimed at controlling the HLB vector. Citrus Ind..

[12] Qureshi J.A., Kostyk B.C., Stansly P.A. (2014). Insecticidal suppression of Asian citrus psyllid *Diaphorina citri* (Hemiptera: Liviidae) vector of Huanglongbing pathogens. PLoS ONE.

[13] Gottwald T.R. (2010). Current epidemiological understanding of citrus huanglongbing. Annu. Rev. Phytopathol..

[14] Tiwari S., Mann R.S., Rogers M.E., Stelinski L.L. (2011). Insecticide resistance in field populations of Asian citrus psyllid in Florida. Pest Manag. Sci..

[15] Tiwari S., Stelinski L.L., Rogers M.E. (2012). Biochemical basis of organophosphate and carbamate resistance in Asian citrus psyllid, *Diaphorina citri*. J. Econ. Entomol..

[16] Hall D.G., Nguyen R. (2010). Toxicity of pesticides to *Tamarixia radiata*, a parasitoid of the Asian citrus psyllid. BioControl.

[17] Michaud J.P. (2004). Natural mortality of Asian citrus psyllid (Homoptera: Psyllidae) in central Florida. Biol. Control.

[18] Subandiyah S., Nikoh N., Sato H., Wagiman F., Tsuyumyu S., Fukatsu T. (2000). Isolation and characterization of two entomopathogenic fungi attacking *Diaphorina citri* (Homoptera, Psylloidea) in Indonesia. Mycoscience.

[19] Xie P.H., Su C., Lin Z.G. (1988). A preliminary study on an entomogenous fungus [*Verticillium lecanii*] of *Diaphorina citri* Kuwayama (Hom.: Psyllidae). Chin. J. Biol. Control.

[20] Hoy M., Singh R., Rogers M.E. (2010). Evaluations of A novel isolate of *Isaria fumosorosea* for control of the Asian citrus psyllid, *Diaphorina citri* (Hemiptera: Psyllidae). Fla. Entomol..

[21] Stauderman K., Avery P., Aristizabal L., Arthurs S. (2012). Evaluation of *Isaria fumosorosea* (Hypocreales: Cordycipitaceae) for control of the Asian citrus psyllid, *Diaphorina citri* (Hemiptera: Psyllidae). Biocontrol Sci. Technol..

[22] Avery P.B., Hunter W.B., Hall D.G., Jackson M.A., Powell C.A., Rogers M.E. (2009). *Diaphorina citri* (Hemiptera: Psyllidae) infection and dissemination of the entomopathogenic fungus *Isaria fumosorosea* (Hypocreales: Cordycipitaceae) under laboratory conditions. Fla. Entomol..

[23] Avery P.B., Wekesa V.W., Hunter W.B., Hall D.G., McKenzie C.L., Osborne L.S., Powell C.A., Rogers M.E. (2011). Effects of the fungus *Isaria fumosorosea* (Hypocreales: Cordycipitaceae) on reduced feeding and mortality of the Asian citrus psyllid, *Diaphorina citri* (Hemiptera: Psyllidae). Biocontrol Sci. Technol..

[24] Hunter W.B., Avery P.B., Pick D., Powell C.A. (2011). Broad spectrum potential of *Isaria fumosorosea* on insect pests of citrus. Fla. Entomol..

[25] Hall D.G., Shatter R.G., Carpenter J.E., Shapiro J.P. (2010). Research toward an artificial diet for adult Asian citrus psyllid. Ann. Entomol. Soc. Am..

[26] Skelley L.H., Hoy M.A. (2004). A synchronous rearing method for the Asian citrus psyllid and its parasitoids in quarantine. Biol. Control.

[27] Meyer J.M., Hoy M.A., Boucias D.G. (2008). Isolation and characterization of an *Isaria fumosorosea* isolate infecting the Asian citrus psyllid in Florida. J. Invertebr. Pathol..

[28] Meitkiewski R., Gorski R. (1995). Growth of selected entomopathogenic fungi species and isolates on media containing insecticides. Acta Mycol..

[29] Asi M.R., Bashir M.H., Afzal M., Ashfaq M., Sahi S.T. (2010). Compatibility of entomopathogenic fungi, *Metarhizium anisopliae* and *Paecilomyces fumosoroseus* with selective insecticides. Pak. J. Bot..

[30] Gupta P., Paul M.S., Sharma S.N. (1999). Studies on compatibility of white muscardine fungus *Beauveria bassiana* with neem products. Indian Phytopathol..

[31] McKenzie C., Kumar V., Palmer C.L., Oetting R.D., Osborne L.S. (2014). Chemical class rotation for control of *Bemisia tabaci* (Hemiptera: Aleyrodidae) on poinsettia and their effect on cryptic species population composition. Pest Manag. Sci..

[32] Cuthbertson A.G.S., Collins D.A. (2015). Tri-Tek (petroleum horticultural oil) and *Beauveria bassiana*: Use in eradication strategies for *Bemisia tabaci* Mediterranean species in UK glasshouses. Insects.

[33] Xu C.F., Xia Y.H., Li K.B., Ke C., Timmer L.W., Garnsey S.M., Navarro L. (1988). Further study of the transmission of citrus huanglongbing by a psyllid, *Diaphorina citri* Kuwayama. Proceedings of the 10th Conference of the International Organization of Citrus Virologists.

[34] Roistacher C.N., Roistacher C.N. (1991). Techniques for biological detection of specific citrus graft transmissible diseases. Graft-Transmissible Diseases of Citrus.

[35] Rogers M.E., Stansly P.A. Biology and Management of the Asian Citrus Psyllid, *Diaphorina citri* Kuwayama, in Florida Citrus. http://www.crec.ifas.ufl.edu/extension/greening/PDF/BiologyandManagementofACP.pdf.

